# Immune checkpoint inhibitors plus chemotherapy in the first-line treatment of young unresectable gastric cancer patients: a multicentre real-world study

**DOI:** 10.3389/fonc.2025.1476402

**Published:** 2025-04-11

**Authors:** Yingnan Wang, Yan Li, Zheng Liu, Jing Liu, Hongmei Xu, Ruixing Zhang, Fengbin Zhang, Zhanjun Guo

**Affiliations:** ^1^ Department of Gastroenterology and Hepatology, The Fourth Hospital of Hebei Medical University, Shijiazhuang, China; ^2^ Department of Oncology, The Handan Central Hospital, Handan, China; ^3^ Department of Cardiovascular, The Fourth Hospital of Hebei Medical University, Shijiazhuang, China; ^4^ Department of Oncology, The Qinhuangdao First Hospital, Qinhuangdao, China; ^5^ Department of Rheumatology, The Fourth Hospital of Hebei Medical University, Shijiazhuang, China

**Keywords:** young gastric cancer, clinicopathological features, PD-1 inhibitors, triple regimens, prognostic analysis

## Abstract

**Background:**

PD-1 inhibitors combined with chemotherapy have become the standard first-line treatment for advanced gastric cancer (GC), but their efficacy in young GC patients is unknown. This study aimed to evaluate the efficacy of immunotherapy in young GC patients and explore new treatment strategies for this population.

**Methods:**

Clinicopathological data of young unresectable GC patients were collected from multiple centres. We defined young as ≤45 years. Statistical analyses were conducted with SPSS IBM for Windows version 24.0.

**Results:**

In total, 225 young unresectable GC patients were registered. Their clinicodemographic characteristics included female predominance (60.9%), poor differentiation (86.7%), high family history of cancer (14.2%), low HER2 expression (12.2%), PD-L1 expression (43.0%) and mismatch repair (MMR) deficiency (1.0%), and a high proportion of peritoneal metastasis (49.3%). After screening, 134 patients were included for analysis: 63 received dual chemotherapy (mFOLFOX6, XELOX, SOX and two-drug containing paclitaxel), 32 PD-1 inhibitors plus dual chemotherapy (mFOLFOX6, XELOX, SOX and two-drug containing paclitaxel), and 39 triple regimens (two-drug chemotherapy combined with apatinib or trastuzumab, or triple chemotherapy based on platinum, fluorouracil and paclitaxel). The effectiveness analysis revealed no significant difference in the disease control rate (DCR) between the dual chemotherapy group and the PD-1 inhibitor plus dual chemotherapy group (*P*=0.787), but triple regimens led to the best DCR (71.4% *vs*. 68.8% *vs*. 94.9%, all *P*<0.05). Kaplan–Meier curves showed median progression-free survival (PFS) times of the three groups of 4.7, 4.7 and 9.2 months, respectively. The median overall survival (OS) was 13.9, 11.0 and 15.9 months, respectively. Multivariate analyses showed that triple regimens were an independent prognostic factor for PFS [hazard ratio (HR) 0.430, 95% confidence interval (CI) 0.263-0.700; *P*=0.001]. Detailed survival analysis demonstrated that patients receiving intraperitoneal infusion of paclitaxel followed by intravenous paclitaxel combined with S-1 and apatinib oral therapy had better PFS (*P*=0.014) and OS (*P*=0.013) than those receiving other regimens.

**Conclusion:**

Young patients with GC have unique clinical characteristics and are not sensitive to immunotherapy. Triple regimens, especially intraperitoneal infusion of paclitaxel followed by intravenous paclitaxel combined with S-1 and apatinib oral therapy, deserve to be studied as first-line therapies.

## Introduction

In 2022, gastric cancer (GC) became the fifth most common cancer and the fifth leading cause of cancer-related death worldwide (1). Although the incidence and mortality of GC have declined over several decades in most parts of the world, the disease burden of GC remains high in Asia. In China, GC is the fifth most frequently diagnosed cancer and the third leading cause of cancer-related death (2). GC typically occurs in middle-aged and elderly patients, especially those aged ≥65 years, but can occur in young patients, although it is largely ignored in the clinic. Recent findings indicate an increasing trend in GC incidence rates among young patients. At present, there is no clear age limit for young patients with GC. In some studies, young GC is defined as GC before age 40, while in others, the definition has included all patients diagnosed before age 45 (3–6). Whether the cut-off is 40 or 45 years, the incidence of young GC patients has increased annually (7, 8). The occurrence of GC in young patients is less affected by environmental factors and more strongly related to heredity (9, 10). Compared with elderly patients, young patients commonly have more aggressive tumours, more advanced disease at presentation, and worse prognoses (11). Meanwhile, due to the lack of obvious early symptoms and delayed diagnosis, young patients often present at an advanced stage when seeking medical care, resulting in the loss of opportunities for radical surgery (12). Therefore, actively exploring pathological molecular characteristics and advancing precision therapy for this condition are of critical significance.

In recent years, immune checkpoint inhibitors (ICIs) have achieved remarkable success across various cancer types, offering a promising advancement in the treatment of GC. Supported by global multicentre phase III clinical trials, programmed death 1 (PD-1) inhibitors combined with chemotherapy have been approved as the standard first-line therapeutic regimen for advanced GC (13–16). However, current treatment strategies for advanced GC remain undifferentiated by age, and no consensus clinical guidelines specifically address the management of young patients in international practice. A recent study demonstrated that immune responses differ with sex and age, with female and young patients having lower response rates to immunotherapy (17). In another multi-institutional retrospective study including 538 patients with advanced melanoma, the association between age and the response to anti-PD1 therapy was evaluated. The study showed that patients younger than 62 years were less likely to benefit from pembrolizumab treatment, with a disease control rate of 52% compared to 63% in older patients. The investigators also noted that the likelihood of response increased with age and that the odds ratios of progressing on pembrolizumab decreased 13% with every decade of patient age (18). Therefore, the efficacy of immunotherapy in young patients still needs to be clarified.

Our previous study demonstrated that young gastrointestinal patients (aged <45 years) receiving PD-1 inhibitor therapy exhibited significantly suboptimal treatment responses and inferior survival outcomes compared to elderly cohorts (19). In this study, we retrospectively analysed the clinical data of young GC patients aged ≤45 years, summarized their clinicopathological characteristics, and compared the efficacy of different first-line treatment schemes, especially PD-1 inhibitors combined with dual chemotherapy, to further verify the efficacy of immunotherapy in young patients and provide data supporting the realization of precision treatment for young GC patients.

## Methods

### Patient screening

Patients were selected for this multicentre retrospective cohort study through the Hebei Gastric Cancer Collaborative Network Database (http://hbss.suvalue.com/), which collected data from the Fourth Hospital of Hebei Medical University, a large cancer centre in Hebei Province, China, between January 2019 and January 2022. To expand the database and exclude single-centre errors, data from two other research centres (Handan Central Hospital and Qinhuangdao First Hospital) were also collected.

### Inclusion criteria

The inclusion criteria were as follows: (i) age 45 years or younger at diagnosis; (ii) histological diagnosis of adenocarcinoma and a diagnosis of stage IV GC, with GC staging based on the 8th edition of the American Joint Committee on Cancer (AJCC) TNM classification. The exclusion criteria were as follows: (i) age>45 years; (ii) insufficient staging information or uncertainty of distant metastasis; (iii) the coexistence of other malignancies; or (iv) other pathological subtypes of gastric malignancies, such as gastric small cell carcinoma, neuroendocrine carcinoma and lymphoma.

### Data acquisition

The following detailed information on the clinicopathologic characteristics of patients was collected from medical records and follow-up systems: age, sex, family history of tumours, tumour location, degree of tumour, human epidermal growth factor receptor 2 (HER2) status, programmed cell death-ligand 1 (PD-L1) expression, microsatellite status, performance status (PS) score, metastatic sites, number of metastatic lesions, history of childbearing (for female patients), operation type, history of palliative chemotherapy, and laboratory tests, including carcinoembryonic antigen (CEA) and carbohydrate antigen (CA) 19-9. The study protocol was approved by the institutional review board of each participating hospital before initiation of the study (2020KS001). All procedures followed medical ethical standards and were carried out in strict accordance with the Declaration of Helsinki and its amendments and other applicable local laws and regulations. Due to the retrospective nature of this study, informed consent was waived by the ethics committee.

### Treatment response assessment

All patients received enhanced thoracic-abdominal-pelvic computed tomography (CT) scans and/or endoscopy before starting the treatments. The CT image sets were retrieved and reassessed according to the 8th edition of the AJCC staging manual. Patients received palliative chemotherapy according to the recommendation of the guidelines and the patient’s preference. Treatment continued until disease progression, treatment-related adverse events, death or loss to follow-up. The Response Evaluation Criteria in Solid Tumours (RECIST) version 1.1 was used to evaluate the efficacy of chemotherapy. The modified RECIST 1.1 for immune-based therapeutic (iRECIST) was used to evaluate the immunotherapy response. Complete response (CR) was defined as the complete disappearance of the target lesion after chemotherapy. Partial response (PR) was defined as a reduction in the total diameter of each target lesion by 30% or more. Progressive disease (PD) was defined as at least a 20% increase in the sum of the long diameters of all target lesions and an absolute increase of more than 5 mm or the appearance of new lesions. Stable disease (SD) was defined as no change in target lesions. The objective response rate (ORR) was defined as the percentage of patients with a CR or PR among all the treated patients. The disease control rate (DCR) was defined as the percentage of patients who achieved CR, PR and SD.

### Follow-up

All patients were followed up via rehospitalization or re-examinations in the outpatient clinic or by telephone until mortality due to any reason or loss to follow-up. Progression-free survival (PFS) was calculated from the date of starting first-line treatment to the date of disease progression or death, whichever occurred first. Overall survival (OS) was calculated from the date of treatment initiation to the date of patient death or the patient’s last follow-up date.

### Statistical analyses

The SPSS 24.0 software package (SPSS Inc., Chicago, IL, USA) and GraphPad Prism 9 (GraphPad Software, San Diego, California) were used for statistical analyses. Qualitative variables and continuous variables are described as frequencies, percentages, means, standard deviations, and medians. Group comparisons of qualitative variables were performed using Pearson’s chi-squared test or two-sided Fisher’s exact test, while continuous variables were compared by means of Student’s *t* tests. Based on Cohen’s w effect size, we need a minimum sample size of 102 to obtain the statistical difference. The basic characteristics of the patients were evaluated by descriptive analysis. Kaplan–Meier curves were used for analyses of survival outcomes, and the log-rank test was carried out to test for statistical significance. Cox regression was used for multivariate analysis with the forward stepwise method. *P*<0.05 was considered statistically significant, and all reported *P* values are two-sided.

## Results

### Patient characteristics and the selection of first-line treatments

From January 2019 to January 2022, 225 unresectable GC patients aged ≤45 years were included according to the inclusion and exclusion criteria. The characteristics of the patients are summarized in **Table 1**. The median age was 37 years (range, 21-45), with 32 patients (32/225, 14.2%) younger than 30. There was a significant predominance of female patients (137/225, 60.9%), and 126 (126/137, 92.0%) of them had a history of childbirth. Notably, among all the female patients who had a history of childbirth, 52 (52/126, 41.3%) had a history of caesarean section, which suggests that caesarean section may be related to the occurrence of GC. There were 109 patients with a PS score of 0 (109/225, 48.4%) and 116 patients with a PS score ≥1 (116/225, 51.6%). In total, 14.2% (32/225, 14.2%) of the GC patients had a family history of tumours. The highest proportion of tumours were in the gastric body (136/225, 60.4%), followed by the gastric antrum (51/225, 22.7%) and gastric cardia (38/225, 16.9%). The vast majority of patients had poorly differentiated tumours (195/225, 86.7%). HER2 status was examined in 172 patients, with 21 (21/172, 12.2%) showing positive expression (immunohistochemistry, IHC 3+ or IHC 2+, fluorescence *in situ* hybridization, FISH amplification). Data on PD-L1 expression were available for 93 patients, 40 (40/93, 43.0%) showing positive expression (combined positive score, CPS≥1). The proportion of patients with CPS≥5 was 20.4% (19/93, 20.4%), and that with CPS≥10 was 8.6% (8/93, 8.6%). Among the 96 patients whose microsatellite status was evaluated, only 1 (1/96, 1.0%) had deficient mismatch repair, dMMR/microsatellite instability-high, and MSI-H. Among the 225 patients, 47 (47/225, 20.9%) patients received palliative surgery, including palliative gastric resection (partial or total) and ovarian resection. There were 49.3% (111/225, 49.3%) of patients with peritoneal metastases at diagnosis.

**Table 1:** Clinicopathological characteristics of young unresectable gastric cancer patients aged ≤45 years (n=225).

Characteristics	n	%
Age (years)		
<30	32	14.2
≥30	193	85.8
Sex		
Male	88	39.1
Female (history of childbirth)	137 (126)	60.9 (92.0)
Familial history of tumor		
Yes	32	14.2
No	193	85.8
HER2 status		
Positive	21	12.2
Negative	151	87.8
Unknown	53	23.5
PD-L1 expression		
Positive	40	43.0
Negative	53	57.0
Unknown	132	58.7
Microsatellite status		
dMMR/MSI-H	1	1.0
pMMR/MSS	95	99.0
Unknown	129	57.3
Tumor location		
Gastric cardia	38	16.9
Gastric body	136	60.4
Gastric antrum	51	22.7
Histological classification		
Highly to-moderately differentiated	30	13.3
Poorly differentiated	195	86.7
Palliative surgery		
Yes	47	20.9
No	178	79.1
PS score		
0	109	48.4
≥1	116	51.6
Peritoneal metastasis		
Yes	111	49.3
No	114	50.7

HER2, human epidermal growth factor receptor 2; PD-L1, programmed cell death-ligand 1; dMMR, deficient mismatch repair; MSI-H, microsatellite instability-high; pMMR, proficient mismatch repair; MSS, microsatellite stability; PS, performance status.

**Figure 1:**
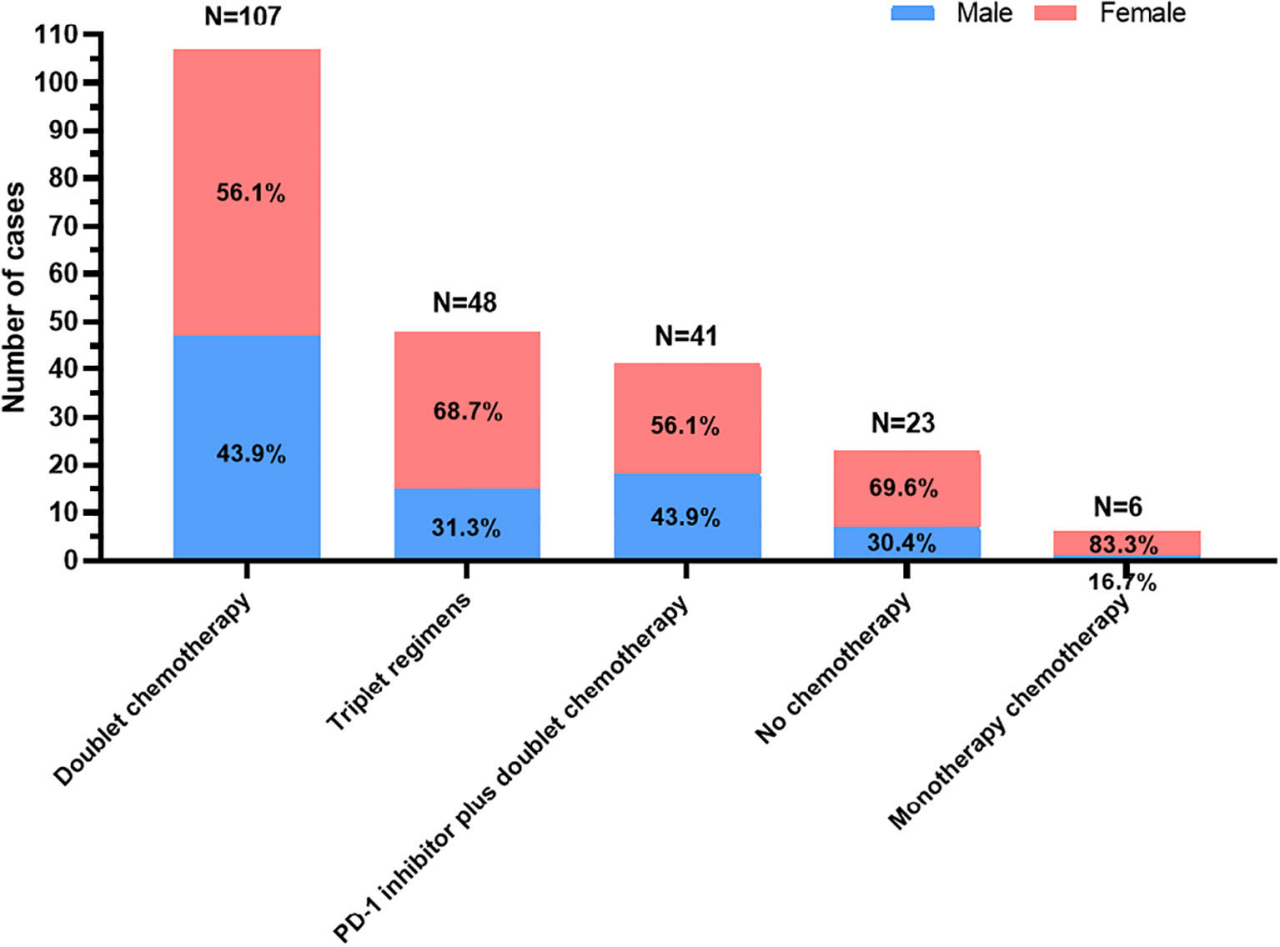
The selection of first-line treatments for young unresectable gastric cancer patients stratified by sex.

Among the 225 patients, 202 received first-line chemotherapy. Among them, 6 patients received monotherapy chemotherapy based on fluorouracil and paclitaxel; 107 received dual chemotherapy based on platinum, fluorouracil, and paclitaxel (mFOLFOX6, XELOX, SOX and two-drug containing paclitaxel); 41 received a PD-1 inhibitor (nivolumab, pembrolizumab, tislelizumab, camrelizumab, toripalimab, or sintilimab) plus dual chemotherapy (mFOLFOX6, XELOX, SOX and two-drug containing paclitaxel); and 48 received triple regimens including two-drug chemotherapy combined with apatinib or trastuzumab, intraperitoneal infusion of paclitaxel followed by intravenous paclitaxel combined with S-1 and apatinib oral therapy or triple chemotherapy based on platinum, fluorouracil and paclitaxel. The selection of first-line treatments for patients stratified by sex is shown in **Figure 1**.

### The efficacy of different first-line therapy regimens in young GC patients

**Figure 2:**
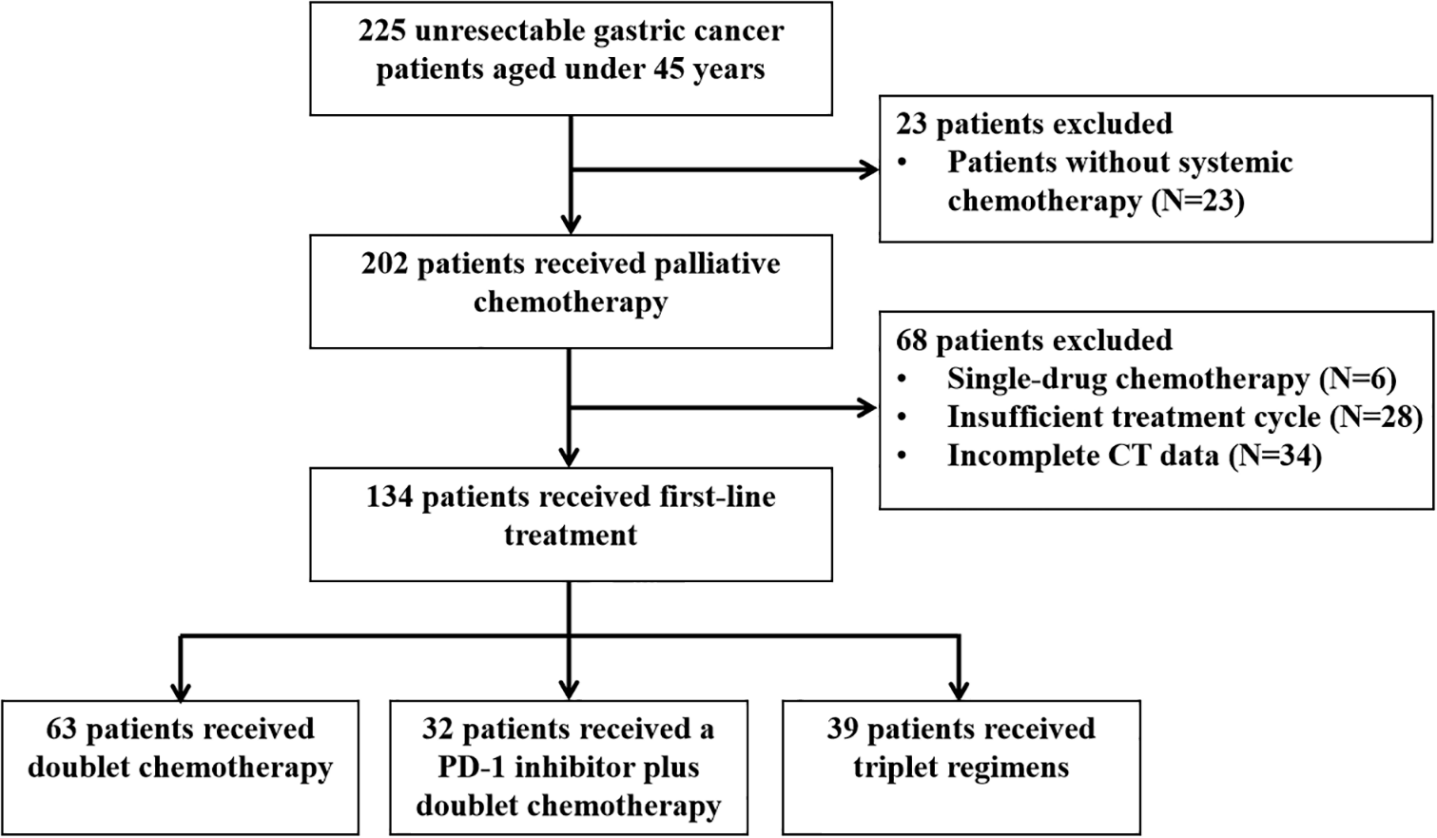
The study cohort selection procedure.

After screening, 134 patients who received at least two cycles of chemotherapy as first-line therapy and had complete data were reviewed. The selection procedure of the study cohort is shown in **Figure 2**. Among them, 63 received dual chemotherapy, 32 received a PD-1 inhibitor plus dual chemotherapy, and 39 received triple regimens. As shown in **Table 2**, compared with the PD-1 inhibitor plus dual chemotherapy and triple regimens groups, the proportion of undetected PD-L1 expression was significantly higher in the dual chemotherapy group (*P*=0.011). Meanwhile, there were more patients in the triplet regimen group that did not receive later-line treatment than in the other two groups (*P*=0.018). Other baseline characteristics, such as age, sex, tumour location, histological classification, PS score, peritoneal metastasis, number of metastatic lesions, familial history of tumour, history of palliative surgery, and CEA and CA19-9 levels at the initiation of first-line therapy, were not significantly different among the three groups.

**Table 2:** The baseline characteristics of young unresectable gastric cancer patients in different treatment groups (n=134).

Characteristics	Total No. (%)	Doublet regimens No. (%)	PD-1 inhibitor plus doublet regimens No. (%)	Triplet regimens No. (%)	*p value ^a^*
Age (years)					0.669
<30	21(15.7)	8 (12.7)	6 (18.8)	7 (17.9)	
≥30	113(84.3)	55 (87.3)	26 (81.3)	32 (82.1)	
Sex					0.593
Male	57(42.5)	28 (44.4)	15 (46.9)	14 (35.9)	
Female	77(57.5)	35 (55.6)	17 (53.1)	25 (64.1)	
Tumor location					0.826
Gastric cardia	24(17.9)	10 (15.9)	9 (28.1)	5 (12.8)	
Gastric body	78(58.2)	43 (68.3)	14 (43.8)	21 (53.8)	
Gastric antrum	32(23.9)	10 (15.9)	9 (28.1)	13 (33.3)	
Histological classification					0.400
High-to-moderate differentiation	15(11.2)	9 (14.3)	4 (12.5)	2 (5.1)	
Poor differentiation	119(88.8)	54 (85.7)	28 (87.5)	37 (94.9)	
PS score					0.976
0	90(67.2)	42 (66.7)	22 (68.8)	26 (66.7)	
≥1	44(32.8)	21 (33.3)	10 (31.3)	13 (33.3)	
PD-L1 expression					0.011
Positive	28(47.5)	7 (11.1)	13 (40.6)	8 (27.6)	
Negative	31(52.5)	15 (23.8)	7 (21.9)	9 (31.0)	
Unknown	75(56.0)	41 (65.1)	12 (37.5)	12 (41.4)	
Peritoneal metastasis					0.088
Yes	66(41.8)	27 (42.9)	14 (43.8)	25 (64.1)	
No	68(58.2)	36 (57.1)	18 (56.3)	14 (35.9)	
Number of metastatic lesions					0.790
1	52(38.8)	23 (36.5)	14 (43.8)	15 (38.5)	
≥2	82(61.2)	40 (63.5)	18 (56.3)	24 (61.5)	
Familial history of tumor					0.826
Yes	17(12.7)	7 (11.1)	5 (15.6)	5 (12.8)	
No	117(87.3)	56 (88.9)	27 (84.4)	34 (87.2)	
Palliative surgery					0.791
Yes	33(24.6)	14 (22.2)	8 (25.0)	11 (28.2)	
No	101(75.4)	49 (77.8)	24 (75.0)	28 (71.8)	
Later-line treatment of tumors					0.018
Yes	100(74.6)	53 (84.1)	24 (75.0)	23 (59.0)	
No	34(25.4)	10 (15.9)	8 (25.0)	16 (41.0)	
CEA level ^b^					0.830
≤1.96 ng/mL	67(50.0)	31 (49.2)	15 (46.9)	21 (53.8)	
>1.96 ng/mL	67(50.0)	32 (50.8)	17 (53.1)	18 (46,2)	
CA19-9 level ^b^					0.686
≤18.08 U/mL	67(50.0)	34 (54.0)	15 (46.9)	18 (46.2)	
>18.08 U/mL	67(50.0)	29 (46.0)	17 (53.1)	21 (53.8)	

PS, performance status; PD-L1, programmed cell death-ligand 1; CEA, carcinoembryonic antigen; CA19-9, carbohydrate antigen (CA) 19–9; PD-1, programmed death 1.

^a^ Chi-square test, or exact chi-square test if any expected cell size <5.

^b^ Take the median of this cohort as the cutoff value.

The efficacy of different first-line therapy regimens in young unresectable GC patients was evaluated according to RECIST 1.1 and iRECIST. As shown in **Table 3**, the ORR of the dual chemotherapy group was 15.9% (10/63), that of the PD-1 inhibitor plus dual chemotherapy group was 12.5% (4/32), and that of the triple regimen group was 23.1% (9/39). The triple regimen group had an advantage in ORR, although the difference was not significant in the current small sample of patients (all *P*>0.05). The DCRs of the three groups were 71.4% (45/63), 68.8% (22/32) and 94.9% (37/39), respectively. There was no significant difference between the dual chemotherapy group and the PD-1 inhibitor plus dual chemotherapy group (*P*=0.787); however, the DCR was significantly higher in the triple regimen group than in the other two groups (all *P*<0.05).

**Table 3:** The clinical response to different first-line therapy regimens in young unresectable gastric cancer patients.

Group	ORR (N=23)		*p* value ^b^	DCR (N=104)		*p* value ^b^
	Events (%)	95% CI ^a^		Events (%)	95% CI ^a^	
Doublet chemotherapy	10 (15.9)	6.6%-25.2%	0.895	45 (71.4)	60.0%-82.9%	0.787
PD-1 inhibitor plus doublet chemotherapy	4 (12.5)	0.4%-24.6%	0.252	22 (68.8)	51.8%-85.7%	0.003
Triplet regimen	9 (23.1)	9.2%-36.9%	0.364	37 (94.9)	87.6%-102.1%	0.004

ORR, objective response rate; DCR, disease control rate; CI, confidence interval; PS, performance status; PD-1, programmed death 1.

^a^ 95% confidence interval with the Clopper-Pearson exact method, the true probability of ORR or DCR falls within the interval with 95% probability; ^b^ Chi-square test, or exact chi-square test if any expected cell size <5.

### Clinical outcome analysis in young unresectable GC patients

**Figure 3:**
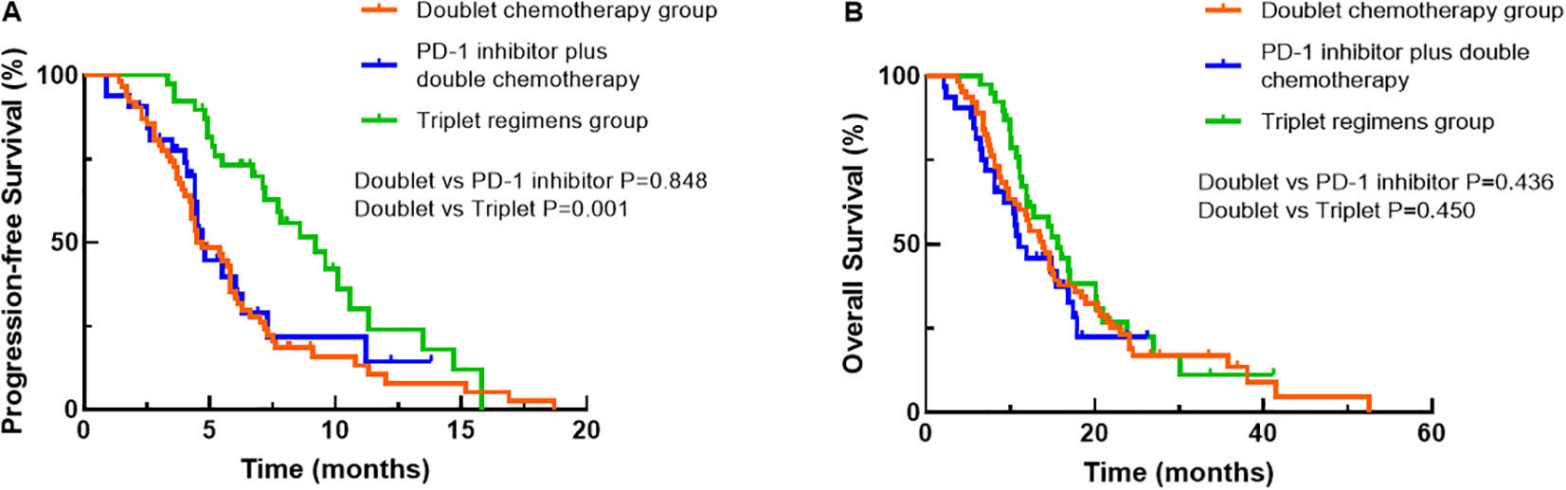
Comparisons of progression-free survival **(A)** and overall survival **(B)** among the treatment groups using Kaplan–Meier curves.

Univariate analysis was performed to find clinical features that might affect the PFS of young unresectable GC patients. The median PFS durations of the three groups were 4.7, 4.7 and 9.2 months, respectively. As shown in **Table 4**, there was no significant difference in PFS between the dual chemotherapy group and the PD-1 inhibitor plus dual chemotherapy group (*P*=0.848, **Figure 3A**), but the patients who received triple regimens had the best PFS (*P*=0.001). Patients with one metastatic lesion had a prolonged PFS compared with those with two or more metastatic lesions (*P*=0.040). In the Cox proportional hazards model, triplet regimens were independently associated with the best PFS [hazard ratio (HR) 0.430, 95% confidence interval (CI) 0.263-0.700, *P*=0.001]. In addition, the number of metastatic lesions (HR 1.604, 95% CI 1.054-2.441, *P*=0.027) was found to be independently associated with PFS (**Table 4**).

**Table 4:** Univariate and multivariate analyses for progression-free survival.

Variable	Univariate analysis		Multivariate analysis		
	HR (95% CI)	*P value*	B	HR (95% CI)	*P value*
Therapy regimens					
Doublet chemotherapy	Reference	0.003		Reference	0.002
PD-1 inhibitor plus doublet chemotherapy	0.951 (0.571-1.586)	0.848	-0.062	0.940 (0.564-1.567)	0.813
Triplet regimen	0.441 (0.271-0.718)	0.001	-0.845	0.430 (0.263-0.700)	0.001
Age (years)					
<30	Reference	0.744			
≥30	1.097 (0.631-1.905)				
Sex					
Male	Reference	0.433			
Female	0.853 (0.573-1.270)				
Tumor location					
Gastric cardia	Reference	0.180			
Gastric body	0.650 (0.379-1.115)	0.117			
Gastric antrum	0.561 (0.296-1.063)	0.076			
Histological classification					
High-to-moderate differentiation	Reference	0.630			
Poor differentiation	0.856 (0.455-1.610)				
PS score					
0	Reference	0.112			
≥1	1.411 (0.923-2.158)				
PD-L1 expression					
Positive	Reference	0.730			
Negative	1.036 (0.562-1.909)	0.911			
Unknown	1.200 (0.709-2.031)	0.497			
Peritoneal metastasis					
Yes	Reference	0.422			
No	1.178 (0.790-1.756)				
Number of metastatic lesions					
1	Reference	0.040		Reference	0.027
≥2	1.551 (1.019-2.359)		0.472	1.604 (1.054-2.441)	
Familial history of tumor					
Yes	Reference	0.931			
No	1.028 (0.548-1.930)				
CEA level ^a^					
≤1.96 ng/mL	Reference	0.589			
>1.96 ng/mL	1.116 (0.750-1.660)				
CA19-9 level ^a^					
≤18.08 U/mL	Reference	0.527			
>18.08 U/mL	0.880 (0.591-1.308)				

PD-1, programmed death 1; PS, performance status; PD-L1, programmed cell death-Ligand 1; CEA, carcinoembryonic antigen; CA19-9, carbohydrate antigen (CA) 19–9; HR, hazard ratio; CI, confidence interval; B, beta coefficient.

^a^ Take the median of this cohort as cut-off value.

Regarding OS, at the data cut-off (September 30, 2022), 103 (76.9%) deaths occurred, 29 (21.6%) patients were alive, and 2 (1.5%) patients were lost to follow-up. The corresponding median OS durations of the three groups were 13.9 months, 11.0 months and 15.9 months, respectively. When OS was compared among the three groups, patients treated with triple regimens tended to have better survival than patients treated with dual chemotherapy and PD-1 inhibitors plus dual chemotherapy, although the difference did not reach significance (all *P*>0.05, **Figure 3B**). Nevertheless, PS score (*P*<0.001), number of metastatic lesions (*P*=0.030) and later-line treatment of tumours (*P*=0.024) were correlated with the OS of young GC patients by univariate analysis (**Table 5**). Based on the outcomes, we conducted further multivariate Cox analysis. The results indicated that PS score (HR 3.791, 95% CI 2.479-5.798, *P*<0.001) and the number of metastatic lesions (HR 1.707, 95% CI 1.116-2.611, *P*=0.014) were independent factors for the OS of young unresectable GC patients (**Table 5**).

**Table 5:** Univariate and multivariate analyses for overall survival.

Variable	Univariate analysis		Multivariate analysis		
	HR (95% CI)	*P value*	B	HR (95% CI)	*P value*
Therapy regimens					
Doublet chemotherapy	Reference	0.417			
PD-1 inhibitor plus doublet chemotherapy	1.224 (0.737-2.033)	0.436			
Triplet regimen	0.835 (0.524-1.332)	0.450			
Age (years)					
<30	Reference	0.809			
≥30	0.937 (0.555-1.583)				
Sex					
Male	Reference	0.279			
Female	1.246 (0.837-1.856)				
Tumor location					
Gastric cardia	Reference	0.413			
Gastric body	0.735 (0.437-1.238)	0.247			
Gastric antrum	0.675 (0.363-1.253)	0.213			
Histological classification					
High-to-moderate differentiation	Reference	0.086		Reference	0.190
Poor differentiation	1.835 (0.917-3.670)		0.472	1.603 (0.791-3.249)	
PS score					
0	Reference	<0.001		Reference	<0.001
≥1	3.768 (2.489-5.704)		1.333	3.791 (2.479-5.798)	
PD-L1 expression					
Positive	Reference	0.950			
Negative	0.929 (0.508-1.698)	0.811			
Unknown	0.919 (0.545-1.551)	0.751			
Peritoneal metastasis					
Yes	Reference	0.314			
No	0.819 (0.555-1.208)				
Number of metastatic lesions					
1	Reference	0.030		Reference	0.014
≥2	1.583 (1.047-2.394)		0.535	1.707 (1.116-2.611)	
Familial history of tumor					
Yes	Reference	0.774			
No	0.912 (0.487-1.708)				
Palliative surgery					
Yes	Reference	0.173			
No	1.390 (0.866-2.234)				
Later-line treatment of tumors					
Yes	Reference	0.024		Reference	0.147
No	1.681 (1.071-2.639)		0.341	1.406 (0.887-2.230)	
CEA level ^a^					
≤1.96 ng/mL	Reference	0.366			
>1.96 ng/mL	0.834 (0.562-1.236)				
CA19-9 level ^a^					
≤18.08 U/mL	Reference	0.799			
>18.08 U/mL	0.951 (0.644-1.404)				

PD-1, programmed death 1; PS, performance status; PD-L1, programmed cell death-Ligand 1; CEA, carcinoembryonic antigen; CA19-9, carbohydrate antigen (CA) 19–9; HR, hazard ratio; CI, confidence interval; B, beta coefficient.

^a^ Take the median of this cohort as cut-off value.

**Figure 4:**
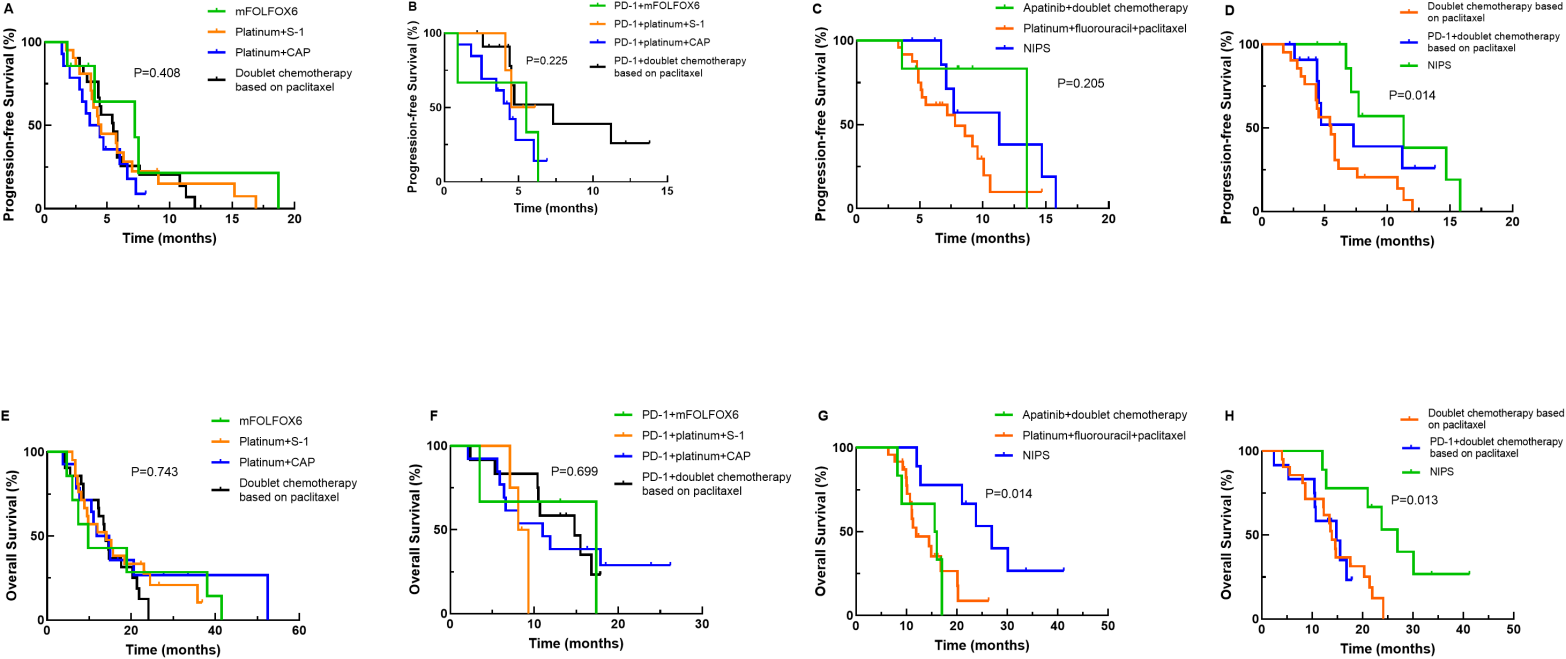
Kaplan–Meier curves for progression-free survival (PFS) and overall survival (OS) stratified by more detailed treatment regimens. **(A)** PFS curves for comparisons between different doublet chemotherapy regimens; **(B)** PFS curves for PD-1 inhibitor combined with different doublet chemotherapy regimens; **(C)** PFS curves for various triplet regimens; **(D)** PFS curves for the neoadjuvant intraperitoneal and systemic chemotherapy (NIPS) group, the paclitaxel-based doublet chemotherapy group, and the PD-1 inhibitor combined with paclitaxel-based doublet chemotherapy group; **(E)** OS curves for comparisons between different doublet chemotherapy regimens; **(F)** OS curves for PD-1 inhibitor combined with different doublet chemotherapy regimens; **(G)** OS curves for various triplet regimens; **(H)** OS curves for the NIPS group, the paclitaxel-based doublet chemotherapy group, and the PD-1 inhibitor combined with paclitaxel-based doublet chemotherapy group. Note: mFOLFOX6, oxaliplatin+calcium folinate+5-fluorouracil.

Next, we performed a more detailed survival analysis based on different treatment regimens. As presented in **Figure 4**, PFS and OS did not differ among the different dual chemotherapy groups (mFOLFOX6, XELOX, SOX and two-drug containing paclitaxel) or the PD-1 inhibitor plus different dual chemotherapy groups (mFOLFOX6, XELOX, SOX and two-drug containing paclitaxel). The patients who received intraperitoneal infusion of paclitaxel followed by intravenous paclitaxel combined with S-1 and apatinib oral therapy had a significantly longer OS than patients treated with other triple regimens (*P*=0.014), but the PFS did not differ among the different triple regimens (*P*=0.205). When intraperitoneal infusion of paclitaxel followed by intravenous paclitaxel combined with S-1 and apatinib oral therapy was compared with the two-drug combination of paclitaxel plus PD-1 inhibitor or all two-drug combinations containing paclitaxel, the patients treated with intraperitoneal infusion of paclitaxel followed by intravenous paclitaxel combined with S-1 and apatinib oral therapy had better PFS (*P*=0.014) and OS (*P*=0.013).

## Discussion

The incidence of GC in young individuals has increased steadily in recent decades (7, 8). Previous research has revealed that the detailed clinicopathologic features and prognostic factors of young patients with GC are still debated. Therefore, we conducted the current study to inve**s**tigate a young group of patient under the age of 45 years. In our analyses, female patients constituted a higher proportion of the entire group, with a male-to-female ratio of approximately 1:1.56. Previous reports have shown that this incidence of GC is higher among males and patients of advanced age, but among younger patients, the incidence is higher among females (20–22). Our study supports this finding of a higher proportion of female patients among young patients. The reason for the female predominance among young patients remains unclear. Several studies have suggested that sex hormones, especially oestrogen, may play an important role in the development of GC in young people (23). Zhong et al. showed that oestrogen-related receptors are highly expressed in GC tissues, where they can promote the migration and invasion of cancer cells (24). Harrison et al. found that oestradiol is trophic to GC cell lines *in vitro* and suggested that positivity for oestradiol receptor D5 expression is an independent negative prognostic factor for patients with GC (25). In addition, sex hormones and sex chromosome-related genes are also the main factors driving these differences in immunity (26, 27). The molecular mechanisms underlying sex-based differences in nonresponsiveness to ICIs, such as oestrogen-mediated recruitment of myeloid-derived suppressive cells (MDSCs) and Tregs to the TME, which are known to be involved in resistance to ICIs, have recently been characterized in mouse models (28). Interestingly, our study found that 41.3% of all female patients with a history of childbirth underwent caesarean section, while the rate of caesarean section in China is only 34.9% (29). The relatively high proportion of patients with GC who underwent caesarean section suggests that caesarean section may be related to the occurrence of GC. However, there are currently few studies on the relationship between the medical history of female patients and GC, and further research is necessary to explore this further. In summary, GC can occur in young female patients, especially those who undergo caesarean section, and symptoms suggestive of GC should be investigated aggressively.

Currently, the occurrence and progression of GC are multifactor and multistep processes that are the result of interactions between the environment and heredity. However, the incidence of GC among young patients is less affected by environmental factors and more related to heredity (9, 10). Familial clustering was found in 11.4% of GC patients and was more notable in young patients (30). In our study, 14.2% of GC patients had a family history of tumours, consistent with other reports (6, 31). The most common hereditary GC is hereditary diffuse gastric cancer (HDGC), which is an autosomal dominant genetic syndrome often caused by E-cadherin gene (CDH1) mutation. Decreased or dysfunctional e-cadherin can lead to decreased adhesion between cells, increasing tumour aggressiveness (32, 33). Due to the limited research scope, we did not sequence the exons of tumour tissues. Nonetheless, it is important to carry out regular endoscopic screening for young patients who have a family history of tumours or genetic mutations who are at risk of GC.

In addition, recent studies have shown that young GC patients have a different molecular expression profile than old patients, including a lower frequency of HER2 overexpression and mismatch repair (MMR) deficiency (34, 35). In our cohort, HER2 status was examined in 172 patients, with 12.2% showing positive expression. According to the HER-EAGLE study, only 9.2% HER2 positivity was detected in patients before age 55 (36). This difference from our study may be explained by the study era and geographic variations. Recent studies have shown that HER2 itself regulates the recruitment of tumour-infiltrating immune cells by inducing the expression of chemokine ligand 2 (CCL2) and PD-1 ligands, thereby enhancing the efficacy of immunotherapy (37). The KEYNOTE-811 study showed that pembrolizumab combined with trastuzumab and chemotherapy increased the ORR of patients with HER2-positive advanced GC to 74.4%, which was significantly higher than that of the control group. Based on this study, immunotherapy combined with anti-HER2 therapy plays an important role in the first-line treatment of advanced GC (16). Microsatellite status was detected in 96 patients, with only 1 of them having dMMR/MSI-H in our survey. The low incidence of MMR deficiency in young patients with GC is probably related to the high proportion of diffuse tumours, wherein MSI is less common, and to the fact that genomically stable tumours are usually diagnosed at an earlier age (38). Patients with MMR deficiency tend to generate more neoantigens and are more immunogenic, thus responding better to ICIs (39). At present, MSI status is the most accurate molecular marker for predicting the efficacy of immunotherapy. In addition, PD-L1 expression was evaluated in 93 patients in our study, 43.0% of whom exhibited positive PD-L1 expression. PD-L1 positivity was defined as a combined positive score (CPS) ≥1. Rha SY et al. included a large cohort of 574 GC patients and demonstrated that 67.4% of all patients had a CPS ≥1 (40). Another analysis showed a positive correlation between PD-L1 expression and the CPS and ICIs response in patients with GC (41). PD-L1 expression has rarely been reported in young GC patients, so these results may provide a reference for further exploration. In conclusion, young GC patients may not be good candidates for immunotherapy, so novel therapies involving different approaches are needed.

Recently, based on the phase III clinical trials, PD-1 inhibitors combined with dual chemotherapy have become the standard first-line treatment for advanced GC patients (13–16). However, therapeutic options for GC are not stratified by age worldwide, and young GC patients may not be good candidates for immunotherapy. In a recent study evaluating the relationship between age and immunotherapy in patients with lung adenocarcinoma, the tumour mutation burden (TMB) of patients in the older group (>50 years) was significantly higher than that of patients in the younger group (≤50 years), and the OS of patients receiving immunotherapy was longer in the older group (42). Specific genetic mutations also influence responses to ICIs. Integrating genomic profiles from TCGA and clinical immunotherapy data from MSKCC, Guan R et al. identified age-related mutational disparities: young patient cohorts exhibited higher frequencies of IDH1, BRAF, and ATRX mutations, whereas TP53, TTN, MUC16, and LRP1B mutations were more prevalent in older cohorts (43). Notably, BRAF mutations appeared to confer inferior responses to ICIs, while patients with LRP1B mutations exhibited significantly higher TMB and improved clinical outcomes compared to wild-type counterparts (44, 45). We also previously reported that PD-1 inhibitors therapy has lower efficacy against gastrointestinal cancer in younger patients (aged <45 years) (18). In this study, we focused on young unresectable GC patients, compared the efficacy of different first-line treatment regimens, and found that PD-1 inhibitors plus dual chemotherapy were not superior to dual chemotherapy alone. The low response rate to anti-PD-1 therapy in young GC patients is not only related to the molecular expression profile of young GC patients themselves but also may be due to the higher incidence of young GC in female patients. A recent study revealed that young and female patients accumulate driver mutations in their tumours that are less readily presented by their major histocompatibility complex (MHC) molecules (17), further suggesting that these effects are strong and complementary. Together, current knowledge provides a rationale for the paradigm that immune selection exerts its Toll differently concerning age and sex, with a strong immunoediting effect being observed in young and female patients. In addition, this study found that 49.3% of young GC patients had peritoneal metastasis at the first visit, which is a negative clinical indicator of immunotherapeutic efficacy. In a multicentre biomarker cohort study on the efficacy of nivolumab treatment for GC, the results showed that patients with nonperitoneal metastases had a higher response rate to immunotherapy than those with peritoneal metastases (46). Previous studies have pioneered the integration of machine learning and single-cell sequencing analysis to systematically investigate the mechanisms underlying immunotherapy efficacy in lung adenocarcinoma (47–49). Our future studies will employ scRNA-seq and spatial transcriptomics to map age-stratified immune landscapes. Coupled with deep learning models (50), this will identify novel targets such as exhausted CD8^+^T cell subsets or immunosuppressive CD8^+^ Treg populations, which may underlie the attenuated immunotherapy efficacy in young patients.

However, young patients are in relatively good physical condition, have few complications, have high tumour malignancy, experience rapid disease progression, and need and tolerate more aggressive treatment options. In the present study, we compared the efficacy and prognostic impact of different first-line treatment regimens in young unresectable GC patients. Triple regimens provided a significant DCR and PFS advantage compared with dual chemotherapy and PD-1 inhibitors plus dual chemotherapy. Although baseline imbalances in patient characteristics, temporal variations, and genetic heterogeneity may compromise the generalizability of the findings, this study provided compelling evidence supporting that these young GC patients may benefit from a stronger chemotherapy regimen. Next, we performed a more detailed survival analysis based on different treatment regimens and found that intraperitoneal infusion of paclitaxel followed by intravenous paclitaxel combined with S-1 and apatinib oral therapy had better PFS and OS than other regimens, which may be related to the higher proportion of patients with peritoneal metastasis in this study. For patients with peritoneal metastasis, due to the existence of a plasma-peritoneal barrier, it is difficult for chemotherapeutic drugs with large molecules to penetrate the barrier to act on metastases in the abdominal cavity, so systemic chemotherapy has little effect on patients with peritoneal metastasis. Intraperitoneal perfusion chemotherapy provides a new treatment option for patients with peritoneal metastasis. Meanwhile, young GC patients frequently present as a poorly differentiated or undifferentiated adenocarcinoma, characterized by heightened tumour cell proliferative activity and a larger proportion of cells residing in active phases of the cell cycle. This aggressive proliferative phenotype may render these tumours more sensitive to paclitaxel, a taxane-class chemotherapeutic agent that targets rapidly dividing cells by stabilizing microtubules and disrupting mitotic spindle dynamics, thereby inducing cell cycle arrest and apoptosis (6). PHOENIX series studies in Japan have confirmed that intraperitoneal infusion of paclitaxel followed by intravenous paclitaxel combined with S-1 oral therapy is a safe and feasible treatment method for GC with peritoneal metastasis (51). In addition, apatinib, as a highly selective VEGFR-2 inhibitor, exhibits fewer off-target effects compared to multi-targeted TKIs, enhancing its therapeutic index in GC (52). Therefore, intraperitoneal infusion of paclitaxel followed by intravenous oral paclitaxel combined with S-1 and apatinib may be recommended as a preferred option for young patients. However, higher quality trials, better patient selection, and multicentre randomized controlled trials are needed to support this conclusion. In summary, there is no doubt that young GC patients have fewer complications, better PS, and adaptability to aggressive treatment. Our results may support an argument that is worth verifying in future research that young GC patients should receive more aggressive treatment.

Our study has several limitations. First, this study was retrospective in nature. Second, the relatively small sample size limited the reliability of the conclusions. Incomplete data on molecular markers such as HER2, MMR status and PD-L1 expression, particularly the unexplored association between PD-L1 expression levels and the therapeutic efficacy of immunotherapy in young GC patients, may introduce bias into the study outcomes. Nevertheless, this study revealed critical clinical characteristics of young GC patients, including a female predominance, lower rates of HER-2 positivity, reduced prevalence of PD-L1 expression, diminished proportions of MSI-H/dMMR, and a higher incidence of peritoneal metastasis. Furthermore, our exploration provided real-world data for the precision first-line treatment of young patients. Prospective validation of age-stratified treatment protocols, particularly in immunotherapy trials, is urgently warranted to translate these findings into clinical practice.

## Conclusions

This study revealed important clinicopathological features of young unresectable GC patients, which may lead to a poor response to ICIs treatment in this population. In terms of first-line treatment in young patients, PD-1 inhibitors plus dual chemotherapy were not superior to dual chemotherapy alone, but triple regimens, especially intraperitoneal infusion of paclitaxel followed by intravenous paclitaxel combined with S-1 and apatinib oral therapy, were superior to other regimens. To our knowledge, this is the first study to explore first-line treatment specific to the population of young GC patients. Prospective new studies are needed to provide more information on this subject.

## Data Availability

The original contributions presented in the study are included in the article/supplementary material. Further inquiries can be directed to the corresponding author.
